# A New, Scalable and Low Cost Multi-Channel Monitoring System for Polymer Electrolyte Fuel Cells

**DOI:** 10.3390/s16030349

**Published:** 2016-03-09

**Authors:** Antonio José Calderón, Isaías González, Manuel Calderón, Francisca Segura, José Manuel Andújar

**Affiliations:** 1 Department of Electrical Engineering, Electronics and Automation, University of Extremadura, Avenida de Elvas, s/n, Badajoz 06006, Spain; ajcalde@unex.es (A.J.C.); calgodoy@unex.es (M.C.); 2 Department of Electronic, Computer Science and Automation Engineering, University of Huelva, Escuela Técnica Superior, Crta. Huelva-Palos de la Fra, Palos de la Fra, Huelva 21919, Spain; francisca.segura@diesia.uhu.es (F.S.); andujar@diesia.uhu.es (J.M.A.)

**Keywords:** data acquisition, real-time monitoring, fuel cell, cell voltage, LabVIEW, Arduino

## Abstract

In this work a new, scalable and low cost multi-channel monitoring system for Polymer Electrolyte Fuel Cells (PEFCs) has been designed, constructed and experimentally validated. This developed monitoring system performs non-intrusive voltage measurement of each individual cell of a PEFC stack and it is scalable, in the sense that it is capable to carry out measurements in stacks from 1 to 120 cells (from watts to kilowatts). The developed system comprises two main subsystems: hardware devoted to data acquisition (DAQ) and software devoted to real-time monitoring. The DAQ subsystem is based on the low-cost open-source platform Arduino and the real-time monitoring subsystem has been developed using the high-level graphical language NI LabVIEW. Such integration can be considered a novelty in scientific literature for PEFC monitoring systems. An original amplifying and multiplexing board has been designed to increase the Arduino input port availability. Data storage and real-time monitoring have been performed with an easy-to-use interface. Graphical and numerical visualization allows a continuous tracking of cell voltage. Scalability, flexibility, easy-to-use, versatility and low cost are the main features of the proposed approach. The system is described and experimental results are presented. These results demonstrate its suitability to monitor the voltage in a PEFC at cell level.

## 1. Introduction

Severe efforts and real compromises are taking place in order to reduce both fossil fuel dependence and greenhouse effect emissions. From the scientific and engineering point of view, relevant efforts are being performed since years ago studying about alternative energy sources, enhancement of efficiency, distributed power generation and so on.

Aiming to contribute to reach a sustainable energetic global scenario, polymer electrolyte fuel cells (PEFCs) constitute an important and promising research field with application in many different systems. A PEFC is an electrochemical device which generates electricity from hydrogen and air. It consists of two electrodes, with a Pt catalyst, separated by an electrolyte. An exothermic reaction occurs when hydrogen and oxygen combine to form water. As a result, electric current, heat and water vapour are generated. The two key parts of a PEFC are the stack (set of stacked cells) and balance of plant (BoP). Electrochemical reaction that generates electricity from hydrogen and air takes place in the stack. The BoP function is to make the stack work properly, so that the electrochemical reaction takes place in the best conditions. The BoP comprises three main subsystems: oxidant/cooling, fueling and electrical [1].

High efficiency, modularity, light weight, low operating temperature, non-combustion, zero emissions, quick start-up and easy integration with renewable energy sources (RES) are some of the features which make PEFCs so attractive for both research and commercial fields. The main drawback is its their high cost. However there are plenty of designs, studies and practical implementations already available in the literature [2,3].

The combination of fuel cells with RES like solar photovoltaic and/or wind energy results in hybrid systems with ability to deal with power fluctuations (due to changing environmental conditions) by using hydrogen for long-term energy storage [4].

Hydrogen generation by an electrolyzer and consuming equipment integration with local RES contribute to distributed generation and early introduction of the hydrogen economy [5]. In this sense, several authors assert that hydrogen will constitute a critical energy carrier in the near future [5,6,7]. In the emerging scope of smart grids (SG), fuel cells and electrolyzers are vital elements for hydrogen production and electricity supply [8].

In [9] it is noted that experimental tests and demonstration projects are very useful to study, analyse and validate methods and tools for the management of new technologies mix for sustainable energy production. Diverse scientific and engineering disciplines are involved in PEFC development and applications, so multidisciplinary research is carried out on diverse areas: chemistry, materials, thermodynamics, electrical, instrumentation, control and so on. Some examples of development and application for both stationary and automotive systems are found in [10].

There are numerous R&D projects related with the design of PEFC to overcome the present limitations and to enhance their performance and lifecycle. Some efforts are focused on air feeding [11], hydrogen feeding [12], electrode material [13], high temperature PEFC [14], hydrogen sources [15] and fault detection and prognostics [16,17,18].

The voltage produced in the PE stack cells has a theoretical maximum value determined by the Nernst potential. However, voltage losses occur, which can be divided in these three major types: Activation losses, ohmic losses and mass transport losses [19].

A PE stack is composed of several cells in series, even hundreds of them. Monitoring of the whole stack operation does provide information about the individual state of the cells. Complex processes take place inside them. The malfunction or fault of any cell affects the behaviour of the rest of them and, hence, the joint performance. Thus, a monitoring system to effectively track individual cells is useful and even mandatory.

As stated by Cadet *et al.* [20] prognostics and health management (PHM) of PEFC uses monitoring data collected during normal operations for maintenance purposes. The first stage consists on data acquisition for further data treatment to identify causes of durability and efficiency reduction. Diagnostic methods utilize different parameters for fault detection such as mean cell voltage, voltage oscillations, pressure oscillations, electrochemical noise voltage, time voltage signal and others. In the latter case, starting, transient and steady-state phases are taken into account. State-of-health diagnosis is commonly based on DC voltage and current [21]. Kim *et al.* [22] proposed a neural network-based method for state-of-health diagnosis of single cell PEFC using the output voltage. Thus, accurate measures of cell current and voltage are required. Modelling of PEFC is related with PHM and research on different issues about PEFC. Development of reliable models strongly depends on rich, precise and representative information, especially those based on grey or black-box methods [23]. Utilization of individual cell voltage as parameter for diagnosis of PEFC systems has been reported in [24,25,26,27]. In [27] an online fault diagnosis of PEFC is carried out using a Support Vector Machine as classification tool. An especially designed device is developed to monitor individual cell voltage and to perform the diagnosis algorithms. Such work demonstrates experimentally that single cell voltage is a critical magnitude to be measured in PEFC systems.

Several factors affect the durability and efficiency of PEFC such as ageing, components degradation, impurities, air composition, materials, catalyst, water management, temperature, membrane humidity, heterogeneous distributions operating conditions inside the cell and cell voltage reversal.

Deep or extensive analysis of the diverse parameters that influence PEFC operation is beyond the scope goal of this paper, but the relevance of proper monitoring systems development has been clearly stated.

Continuous monitoring and supervision of the stack cells implies the use of sensors, DAQ devices and software to represent and store the information. Next, some considerations about these elements are exposed.

Measuring and logging diverse parameters is part of the inherent nature of scientific and technologic activities. The term data acquisition (DAQ) is often applied to a variety of measures, ranging from analog to digital signals of several types [28].

As commented above, cell voltage measurement is a fundamental task with a lot of potential applications in PEFC research. In this paper, a new, scalable and low cost multi-channel monitoring system to implement the data gathering of individual cells voltage in a PEFC is presented. An Arduino board, model MEGA2560, plays the role of DAQ system (the cost of Arduino MEGA is noticeably lower than the cost of a traditional DAQ system) whereas a NI LabVIEW program is responsible for data logging and real-time monitoring. Voltage history of all and every one of the cells is registered and supervised. It has a research orientation to be used as a powerful tool in studies about PEFC supervision and life-time enhancement.

The rest of the paper is organized as follows: in the following section a brief review of DAQ and supervisory control and data acquisition (SCADA) architectures is shown. This kind of systems are the start point of the developed system, therefore in order not to make too large the introduction section we have preferred to carry out the review of this systems in a separate section. Description of the developed system is found in Section 3. This section reflects the depth of the work done both hardware and software. Section 4 shows the usefulness of the developed system by means of an application to an actual PFC. Finally, main conclusions and further works are outlined in Section 5.

## 2. A Brief Review of DAQ/SCADA Architectures

Monitoring systems are mainly based on two elements: the DAQ system and the master terminal unit (MTU). The DAQ system is a device connected to the process or system whose variables (digital and/or analog) have to be registered. MTU usually comprises a PC and a software application running which is responsible for reading the variables acquired by the DAQ system. This is usually a microcontroller mounted on a board with signal conditioning circuits integrated. The signal to measure might be directly wired to its input ports or can require the interfacing of a proper sensor to convert the signal to an electrical standardized range. In most cases, there are also some output ports, so commands signals can be applied by the DAQ system.

Depending on the capabilities of the DAQ system, two different configurations can be considered. The first one is the stand-alone option, which implies a DAQ system operating in an autonomous way until the connection to the MTU. To this purpose, local data storage memory and long-term power supply must be included in the resources of the DAQ system. Once linked to the MTU, a software application exchanges information with the DAQ system so data accumulated in its memory are downloaded to the PC memory for further use.

**Figure 1:**
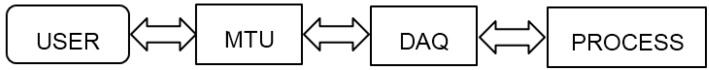
Block diagram of general monitoring system based on DAQ system and MTU.

The second scheme is a computer-based solution which consists on the DAQ system permanently connected to the MTU where the associated software is running, as illustrated in Figure 1. For data flow purpose, a communication channel has to be established between both elements. In general, it is carried out by a wire supporting communication protocol such as RS-232, RS-485 or USB. It is also possible that the DAQ system (contained in a card) is integrated in the PC by means of the PCI bus. Nowadays, wireless links like Wi-Fi, Bluetooth or ZigBee, are receiving increasing attention.

Monitoring of the process is carried out by the software application. It runs in the PC and receives data collected by the DAQ system. Typically, the application handles three main functions: configuration of the DAQ system, data storage and data representation (numerical and/or graphical). In addition, this program can include other useful functions like alarm notification, statistics calculations, report generation and even advanced communication capabilities (object linking and embedding for process control, OPC or OLE for process control). The user is continuously enriched by information from operative status and measurement data.

If the monitoring system is able to generate and apply control commands, we are talking about centralized supervisory platforms, commonly called SCADA systems. These systems display real-time information of the plant (system to monitor and control where the process is taking place) behaviour and store the relevant variables for further analysis and interpretation.

The implementation of a custom-designed monitoring system can be carried out with proprietary or open-source software. Wonderware InTouch [29], IntellutioniFIX [30], Siemens WinCC [31], National Instruments LabVIEW [32] are some examples of proprietary packages. On the other hand, RapidSCADA [33], OpenSCADA [34], IndigoSCADA [35] represent open-source alternatives.

There is no an absolute best option in the decision of the proper software. To simplify, open-source packages require advanced programming skills whereas proprietary software can be expensive. Depending on the particular cases, each one of them will better satisfy the requirements. In our case, we have chosen proprietary software, NI Laboratory Virtual Instrumentation Engineering Workbench (LabVIEW), with worldwide presence and support for thousands of technologies and instruments [36]; in fact we could say that is practically the standard in the professional world of instrumentation. On the other side, an open-source hardware platform has been selected for the DAQ role.

Monitoring and SCADA systems play a paramount role in the optimal operation of the systems under control and supervision. An important requirement of SCADA and monitoring systems is flexibility to adapt to new situations and equipment, particularly due to the constant technological changes that take place in the renewable energy field [37].

In [38] SCADA and monitoring systems are considered an essential infrastructure to evolve towards an energetic system based on decentralized generation. Authors emphasize that these systems have to be designed to act as the core of smart grids (SGs), the grid of the future. In fact, the capability of monitoring everything from power plants to consumer preferences is one of the main characteristics of SGs [39].

According to Sung and Chung [40], monitoring systems applied to energy scope have recently experienced growth due to the environmental awareness and energy shortages. The authors assert that intelligent monitoring is indispensable for RES.

Recent trends in monitoring and SCADA systems are related with e-maintenance [41], open connectivity [42], cyber security [43], cloud data storage [44], smart metering infrastructure [45,46], internet of things [47] and machine-to-machine communication (M2M) [48].

Regarding DAQ choice, it is relevant to state that different open-source microcontrollers and microprocessor platforms have arisen during last years. The most illustrative examples are Arduino [49], RaspberryPi [50], Intel Edison [51] and openDAQ [52]. These devices provide easy-to-use hardware and software to develop multiple projects.

Specifically, Arduino is a low-cost single-board microcontroller which acts as open-source hardware platform. In recent years, this device has become a versatile and powerful tool to develop different applications in the fields of data acquisition, automation and engineering in general.

Among the numbered options, we have chosen an Arduino board for the present application due to its wide and increasing presence in academic and engineering institutions. Open-source code is one of its advantages. All around the world there are numerous projects and information involving academic, scientific and non-professional available resources.

Microcontroller-based monitoring systems have been developed and proved their suitability for environmental [53], biomedical [54] and industrial applications [55]. In the case of the Arduino board, it has been used for data acquisition purposes in several works; some examples in the field of RES monitoring are described below. In Gad [56] an Arduino MEGA connected to a PC and to a microcontroller acquires signal from temperature sensors in the context of solar energy applications. In other cases, Arduino is utilized for transmission through the ZigBee protocol in the context of wireless sensor networks [57,58,59].

Regarding PEFC-based systems, the NI LabVIEW package has been previously applied. Identification of voltage losses indicators in PEFC stacks was performed using a NI LabVIEW application by Husar *et al.* [19]. Hsieh and Huang [60] developed NI LabVIEW programs to control experiments and generate polarization curves of a planar array micro fuel cell stack. In [61] a monitoring system based on NI LabVIEW was applied to acquire a 200 W stack voltage and other parameters to feed a PEFC model. Moçotéguy *et al.* [62] studied the influence of ageing on PEFC stacks with a NI LabVIEW program to monitor the processes. The same authors in [16] used an identical system to analyse water management faults in PEFC stacks. In [63] a customized application controlled and monitored a test bench for experiments about purging effects on PEFCs. In [64] the use of a NI LabVIEW-based SCADA for an autonomous hybrid renewable energy system including PEFC and electrolyzer is described. Lebreton *et al.* [65] managed variables in an experimental test bench with a SCADA developed with NI LabVIEW to apply fault tolerant control for water management in a single cell PEFC. A current distribution mapping was developed by Lilavivat *et al.* [66] using a NI LabVIEW monitoring program and a set of Hall-effect sensors applied to a custom-designed PEFC. In [27] a strategy for fault diagnosis of PEFC systems based on individual cell voltage was described and applied to a 64-cell stack under real conditions. In that work, a NI LabVIEW-based interface implemented the visualization of cell voltage data and the diagnosis results. In [67,68], NI LabVIEW implemented a SCADA system for simulation and real-time monitoring of a modular PEFC system. The authors indicate that commercial monitoring interfaces are very expensive and lack some important features like openness, ease-of-use and ability to adapt to different PEFC-based systems.

Despite the capabilities of Arduino, it has not been reported as an element for PEFC-related monitoring. Consequently, NI LabVIEW and Arduino integration for PEFC monitoring does not appear in the scientific literature consulted by the authors, so we can conclude that our proposal constitutes a novel application. The only paper reporting a somewhat related application is [69], where an Arduino UNO board is used with NI LabVIEW for monitoring sediment microbial fuel cell electrodes. Out of fuel cell scope, in [70] Arduino MEGA and LabVIEW are combined to handle data acquisition and monitoring in a low-cost platform for characterization of photodetectors.

In previous works, the authors have measured and registered PEFC stack parameters using a SCADA system based on a Siemens s7-300 PLC and an HMI panel [71,72]. A SCADA solution has also been developed via a NI USB DAQ and NI LabVIEW [64].

## 3. Developed Monitoring System

Arduino MEGA is based on a ATmega2560 microcontroller. It has 54 digital inputs/outputs (15 of them can be used as PWM outputs), 16 analog inputs, four UARTs, a 16 MHz crystal oscillator, a USB connection, a power jack, an ICSP header and a reset button.

**Figure 2:**
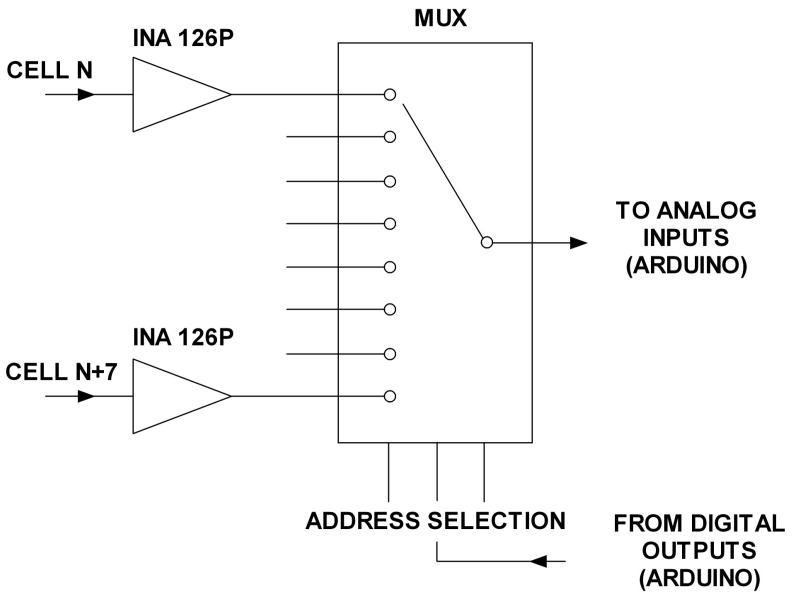
AMB schematic diagram.

**Figure 3:**
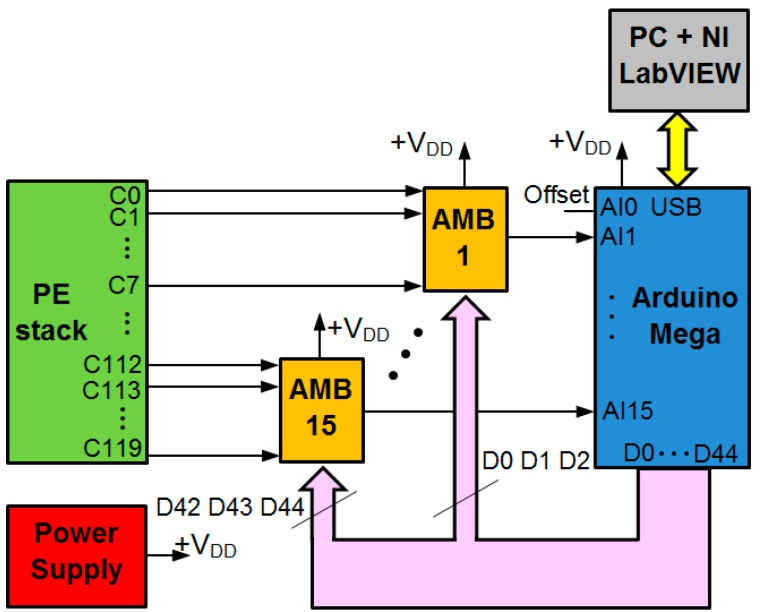
Developed monitoring system architecture.

The monitoring system design has been carried out to fulfill the already mentioned objectives of scalability, flexibility, ease-of-use, versatility and low cost. In this sense, achieving the first objective poses a problem as the number of analog inputs available on Arduino is very low, only 16. This makes it impossible to connect the voltage of each stack cell to a separate input (consider for example that a stack of several kW can have 80 or more cells). Scalability means that the system should be able to easily grow according to the number of required inputs (cells) for each application (each stack). In order to overcome this limitation, an amplifying and multiplexing board (AMB) has been designed and built (Figure 2) as interface to connect the PE stack individual cells to each Arduino input channel. It is composed by eight INA126P instrumentation amplifiers (one per cell) and a multiplexer as main elements. Each AMB provides eight analog input channels, one analog output channel and three digital selection inputs which are governed by three digital Arduino outputs. This way the multiplexer can select which of the eight AMB input channels passes to an Arduino analog input. The input selection (particular cell) is established by the real-time monitoring software (a NI LabVIEW program). Figure 3 shows the developed monitoring system architecture.

Arduino MEGA can be powered via its USB connection or with an external regulated 5 V power supply. In our design the second option was adopted (see Figure 3). Memory available is composed by 256 kB of flash memory for storing code, 8 kB of SRAM and 4 kB of EEPROM. Each of the 54 digital input/output can be configured to be used as an input or output. They operate at 5 V. The 16 analog inputs provide 10 bits of resolution thanks to the 10 bit analog-to-digital converter.

The Arduino integrated development environment (IDE) allows the programming of tasks to perform. It is a cross-platform application written in Java, which can be used for any Arduino board. Arduino-compatible expansion boards, known as shields, can be directly plugged into the standardized pin-headers of the Arduino board. Some examples are GPRS/GSM, RFID, GPS, TFT touch display, Wi-Fi, Ethernet, Bluetooth, CAN bus, pre-assembled sensors, micro SD memory and many others. This provides huge potential for remote monitoring and scalability.

As commented previously, the monitoring system has been designed with the widely known NI LabVIEW package. Applications developed with this software are called Virtual Instruments (VI). Graphical block diagram facilitates configuration in combination with powerful built-in functions. NI LabVIEW Interface for Arduino toolkit performs the communication between both elements for data exchange.

Analog input AI0 of Arduino (see Figure 3) is used for measuring the offset signal (explained in next paragraphs), so the other 15 inputs (channels) are available to measure cell voltages. The multi-channel character of our proposal relays on the existence of one AMB for each of these channels, hence multiplying by eight the input capability. For example, if a stack of 80 cells needs to be monitored, 10 AMB + 10 Arduino MEGA analog inputs will fulfil the requirement.

According to the PEFC specifications, the nominal cell voltage is 0.4–0.96 V. However, fuel starvation can lead to the appearance of negative values, a phenomenon known as cell reversal or null values. Both of them indicate a malfunction of the cell, so the system needs to be able to measure negative voltage values despite the fact that the input range of Arduino is 0–5 V. To overcome this limitation, our solution consists on adding a regulated DC offset to each input channel of the AMB. This signal is measured by the Arduino AI0 analog input. After that, in order to obtain the actual cell voltage, this offset will be subtracted from each measurement in the NI LabVIEW program. When cell reversal occurs, the result of such difference will be lower than the offset. Thus, a means to detect the cell reversal phenomenon is achieved. This situation as we discussed later in this section is exhibited using an alarm LED indicator.

Considering Figure 3 we can think that perhaps we are using too many digital outputs to govern 15 possible AMBs. Of course by using combinational logic (and additional circuitry) the number of Arduino digital outputs necessary to cover the addressing of 120 cells through AMBs could be reduced to 8. However there are plenty of digital outputs in Arduino that will be unused, thus it makes no sense to complicate the architecture (also the programming) and make it more expensive. In any case it is something that could be done at any time. Figure 3 shows the developed monitoring system architecture.

### Real-Time Monitoring System

A software interface has been developed with NI LabVIEW for real-time monitoring of the PE stack cells’ behaviour. Using the built-in functions, data received from Arduino are processed and displayed in a Graphical User Interface (GUI). The GUI affords continuous feedback information. Numerical and graphical representation, refreshed in real-time, allows rapid inspection and evolution tracking of the variables.

NI LabVIEW package is a high-level graphical language that supplies the desired flexibility to make modifications for adapting to device changes or algorithm improvements. As exposed before, data to be represented comes from the Arduino DAQ subsystem. The physical connection with the PC running the GUI consists of a USB cable.

Communication with Arduino is programmed directly with NI LabVIEW using the Arduino toolkit. Basic Arduino Sketch for interfacing with LabVIEW provides support for this task. In this sense, the sketch LIFA Base is executed continuously in the Arduino MEGA to carry out the data exchange with LabVIEW, the polling of input signals and writing of digital outputs. These outputs perform the addressing of the input channel to be read. In other words, Arduino is always looking for commands sent through the serial port from LabVIEW and sampling inputs for obtaining new input data.

**Figure 4:**
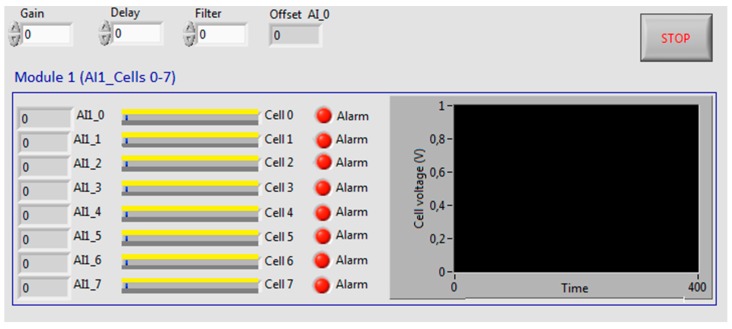
GUI for PEFC voltage tracking.

The GUI is modular and it has been designed to offer a user-friendly environment and intuitive data representation. The first module (cells from 0 to 7) can be seen in Figure 4. Apart from a set of numerical fields and alarm LED indicators, horizontal bars show the voltage of each cell. In addition, a chart shows the evolution of one of the cell voltage. The user can select these options: Sampling time, number of samples for a smoothing software filter and cell voltage to be represented in the chart.

**Figure 5:**
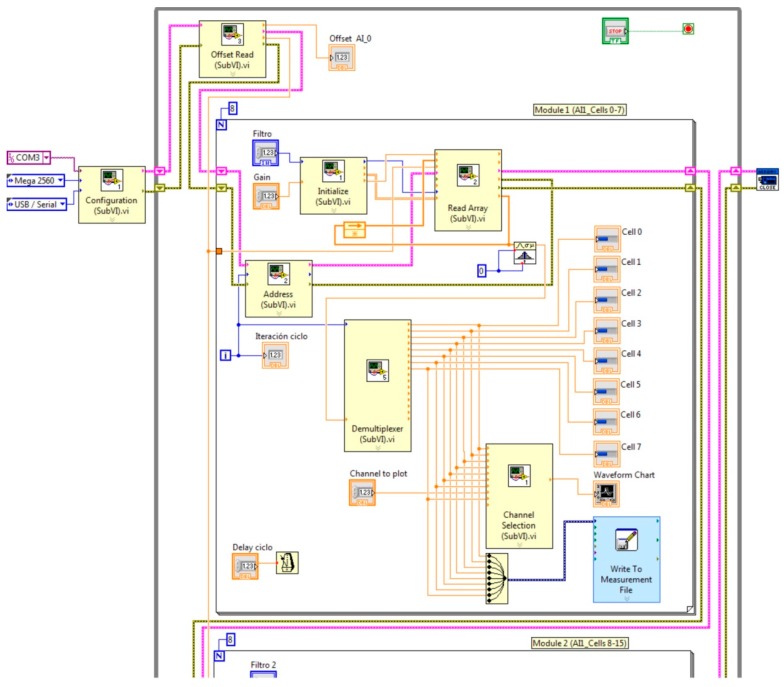
Functions to implement NI LabVIEW interface.

Figure 5 shows the block diagram of the VI that performs the processing and monitoring operations (cells from 0 to 7). As can be seen, a subVI has been designed to implement each function.

**Figure 6:**
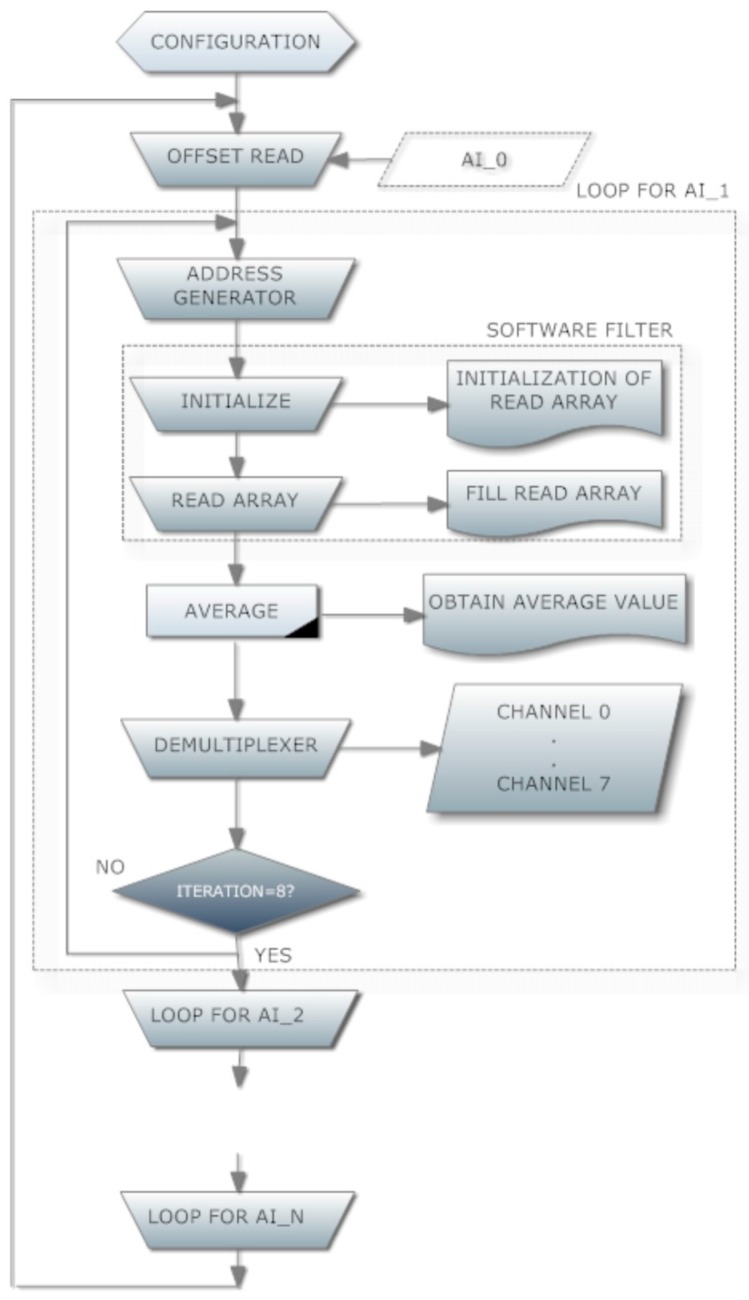
Flowchart of the NI LabVIEW program.

The flowchart of Figure 6 depicts the sequence of operations. The first one is the configuration of communication parameters. Once, this task has been fulfilled, the following step is the dc offset reading. Next, a for loop is executed eight times for each Arduino analog input. This number matches with the AMB input channels. Address for multiplexing AMB inputs is generated in every iteration in order to select a particular cell. For each cell, a software filter calculates an average value to smooth the measurements. For this purpose, an array is created and filled with the aforementioned measurements. Once the mean value has been obtained, a demultiplexing step takes place. This way, the averaged value of each cell voltage is sent to the respective numerical indicators of the interface. Finally, data storage and graphical visualization are performed.

Regarding data logging, it is evident that for mid and long-term investigation and surveillance, acquired data must be saved. This way, a historical data base that delivers rich feedback information is created. Large amounts of data require an effective and secure management system to avoid information loss and facilitate future analysis. In our proposal, data are stored in the form of comma-separated values (.csv) files in the local memory of the PC. In fact, the experimental data presented in the Results section come from such recorded files.

## 4. Discussion

In this section, real time data acquisition and visualization during several experiments are reported. Thus, it has been possible to observe the variables evolution and check the correct working of the fully functional implementation of the system.

**Figure 7:**
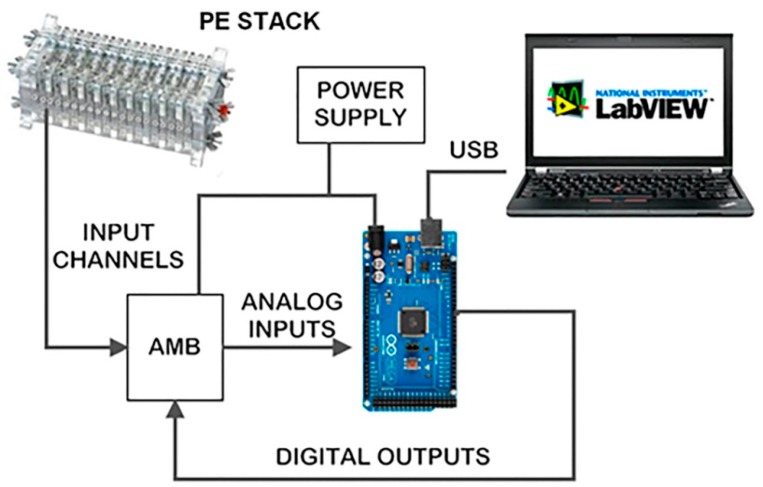
General block diagram of the experimental system.

The monitored PEFC is a low power range and modular PE stack of 10 cells from the manufacturer h-tec [73]. Table 1 contains the main features of the PE stack and Figure 7 shows the block diagram of the experimental system. As can be seen by comparing Figure 3 and Figure 7 the experiment was conducted at the lowest level of the real possibilities of the system (in order to carry it out at low cost). Using system full capacity involves simply replicating 15 times the number of AMBs, as shown in this section. The rest of the architecture remains unchanged (except of course the wiring); it is only a matter of programming from the real-time monitoring subsystem hosted in the PC. This shows clearly the flexibility and scalability of the developed monitoring system.

**Figure 8:**
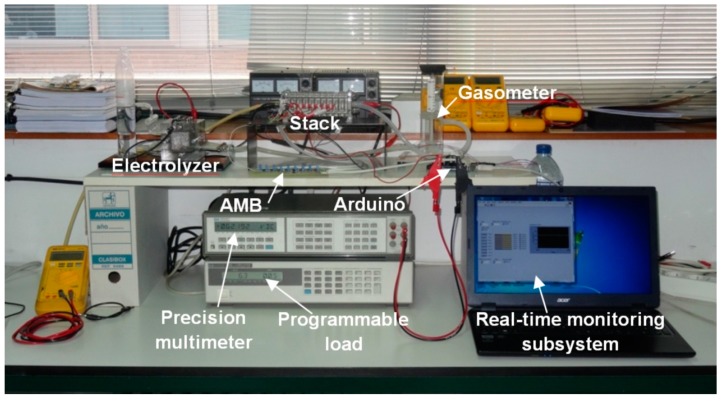
Laboratory setup for experimental tests.

The experimentation has been conducted in the facilities of the Electrical Engineering, Electronics and Automation Department of the University of Extremadura (Spain). Figure 8 illustrates the laboratory setup. Some devices complete the general block diagram of Figure 8: A HP 3457A precision multimeter to validate the measurements, a HP 6063B programmable load to configure load profiles on demand and an electrolyzer to produce the fuel needed, hydrogen of course. Feeding the PE stack does not occur directly from the electrolyzer, this is not practical. A gasometer stores the produced hydrogen until the PE stack uses it.

**Figure 9:**
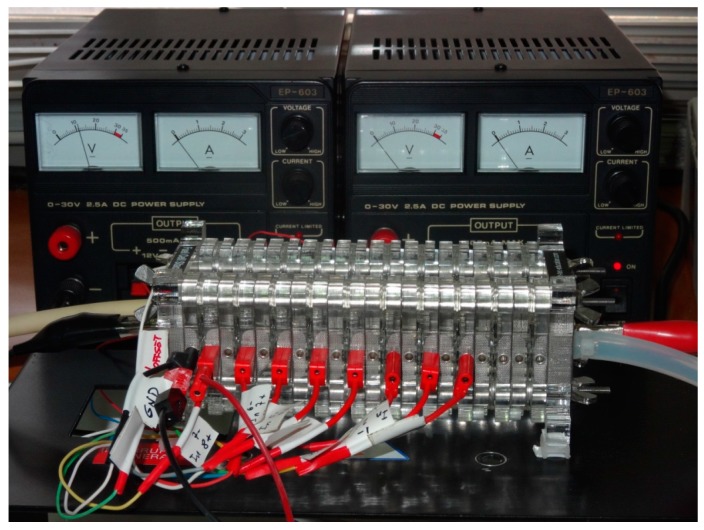
PE stack assembled and wired under laboratory tests.

**Figure 10:**
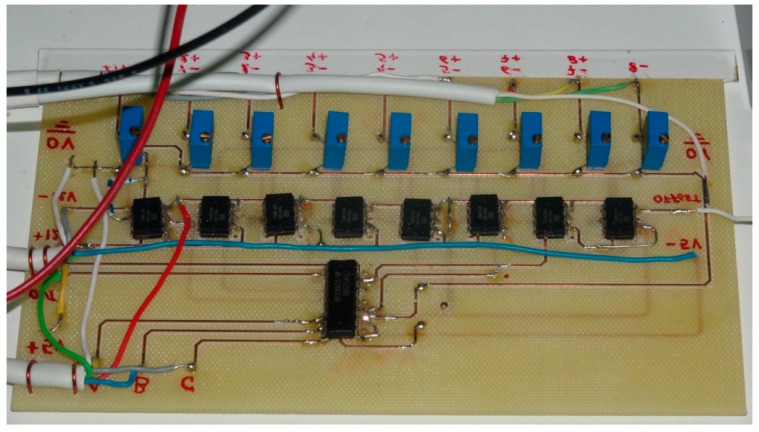
Assembled AMB under laboratory tests.

**Figure 11:**
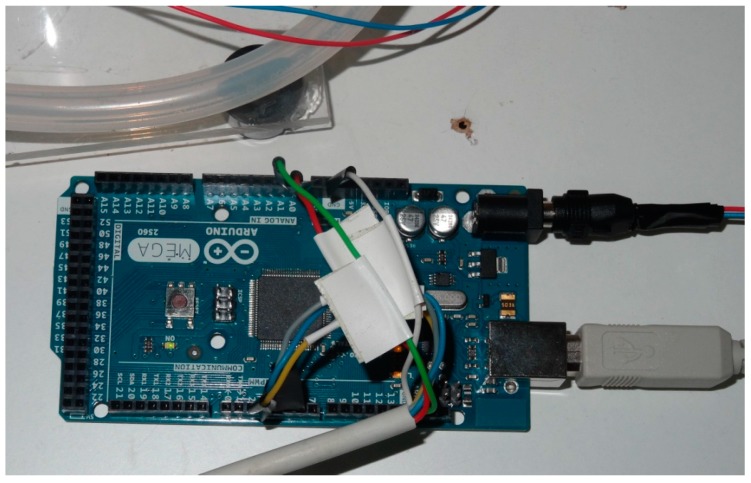
Arduino MEGA connected under laboratory tests.

Voltage measuring sockets are located on one side of each cell. Physical wiring of the whole stack can be observed in Figure 9. AMB is shown in Figure 10, whereas Figure 11 shows the Arduino board connected to the power supply, PC and AMB.

The monitoring system is fully functional so we have driven several tests with different conditions and hours of working to check the stability and reliability. Sample results are provided. Both, open circuit and load connected regimes have been tested.

**Figure 12:**
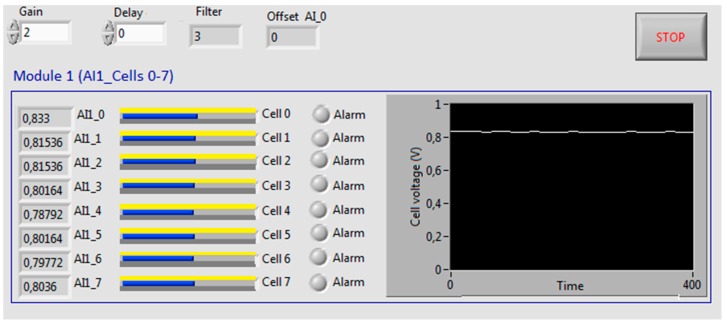
GUI during open circuit test.

In Figure 12, monitoring values obtained during an open circuit test can be observed on the designed GUI. The graphical representation in all the figures corresponds to the first cell of the stack. As can be appreciated, voltage values are stabilized around the same value, 0.8 V, demonstrating a correct working of every cell.

**Figure 13:**
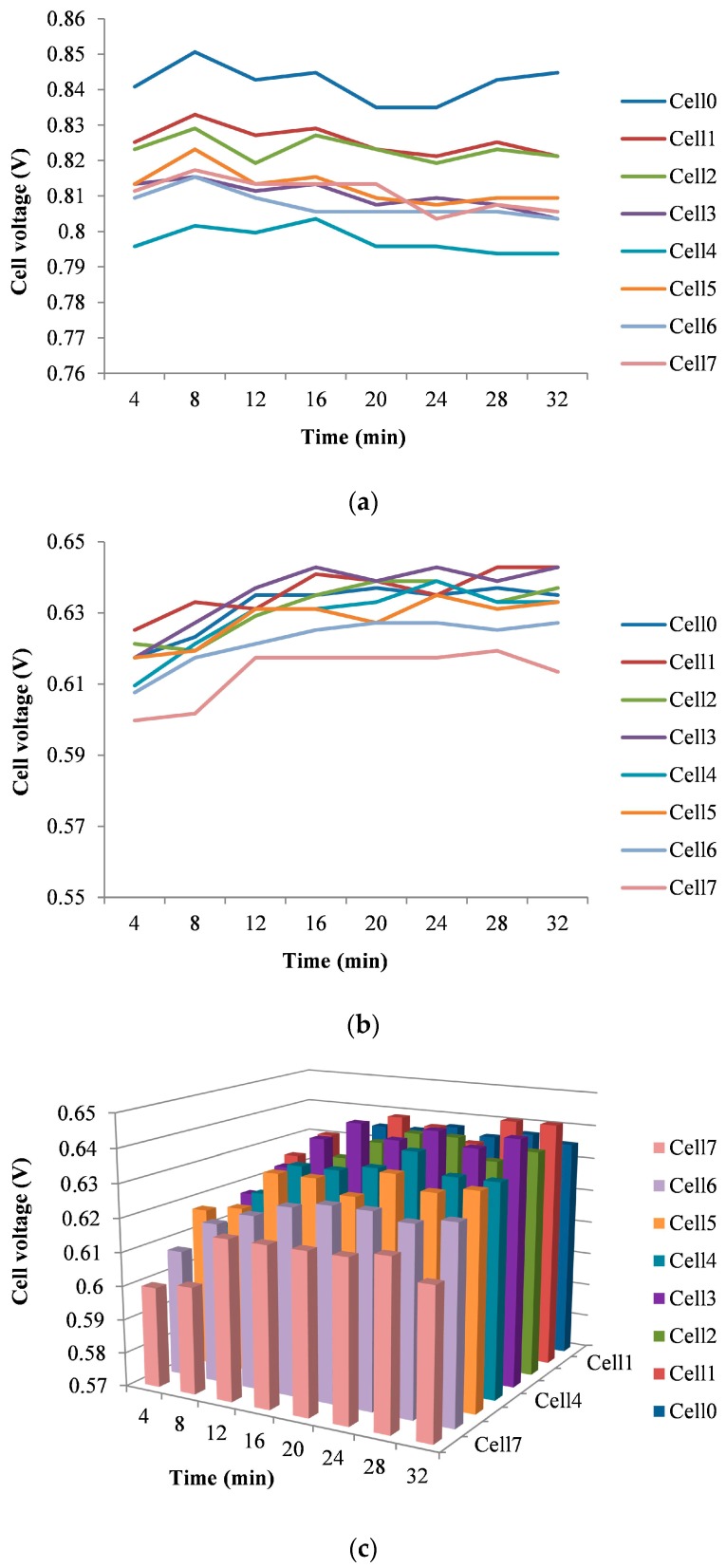
Evolution of each cell voltage: (**a**) Open circuit; (**b**) Connected load; (**c**) Connected load with three-dimensional chart.

Figure 13 shows the voltage evolution of the eight monitored cells for: (a) open circuit; (b) load supply; and (c) load supply with three-dimensional representation. These tests last 30 min. As can be seen, voltages are very stable and inside the expected range.

**Figure 14:**
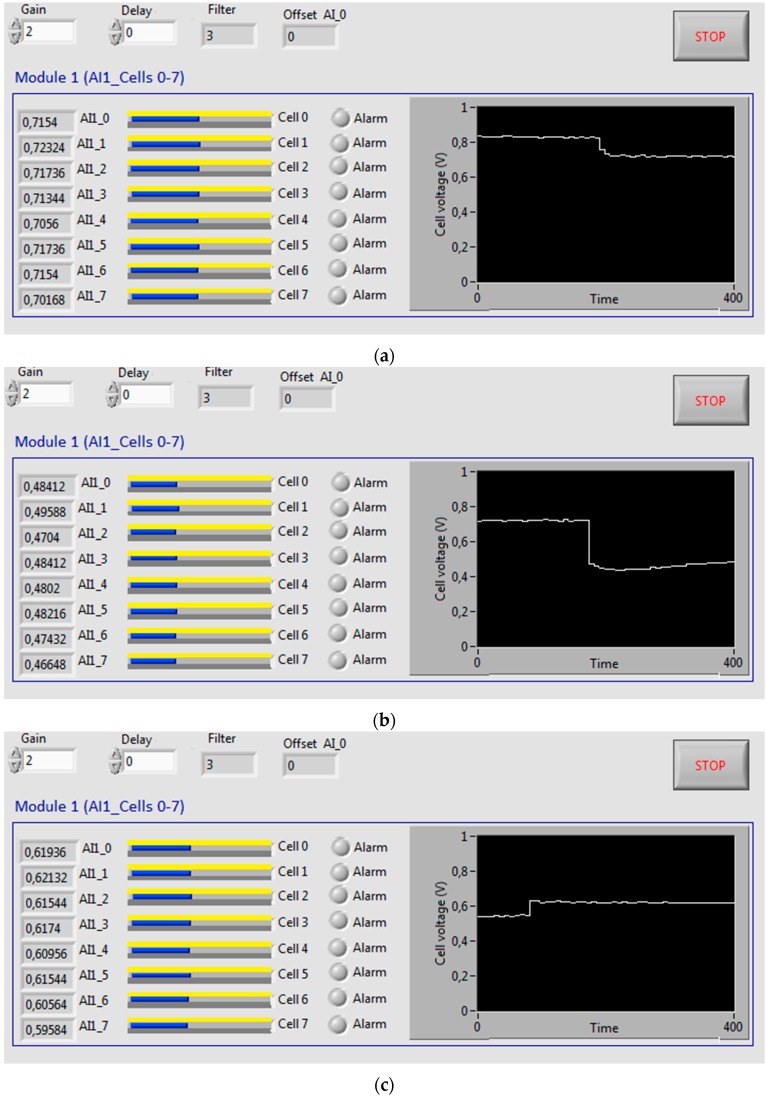

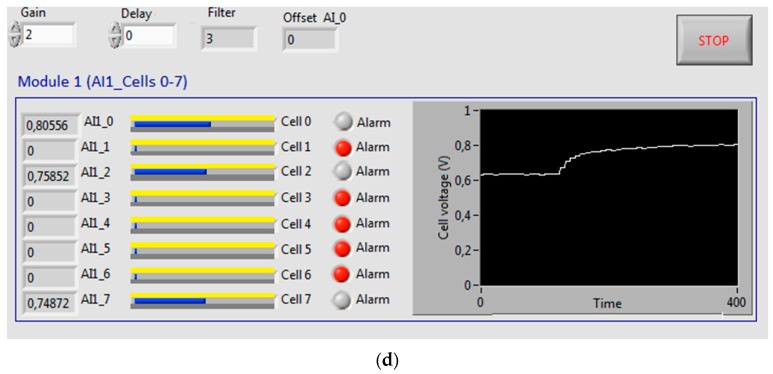
GUI under different operation regimes: (**a**) load connection; (**b**) Increase of load demand; (**c**) Reduction of load demand; (**d**) Alarm situation due to hydrogen scarcity.

In Figure 14 different operation regimes depending on the load and hydrogen supply are reflected. The chart of Figure 14a illustrates the transition between open circuit state and the connection of a load demanding 0.01 A. As a consequence, the voltage of all cells drops to meet the load. The same effect can be observed in Figure 14b when the load demands higher current, from 0.01 to 0.1 A. In this case, the drop in voltage is greater than in the previous one. On the contrary, in Figure 14c the cell response when the demanded current falls from 0.1 to 0.05 A can be observed. As expected, the cell voltage increases. Finally, Figure 14d is very interesting since it corresponds to a shortage of the fuel flow and its heterogeneous distribution. The cells numbered as 0, 3 and 7 have a residual amount of hydrogen. This level is enough to maintain a voltage value but not sufficient to produce current circulation. Here, we can see that the first cell reacts with a progressive recovery of voltage. As the rest of the cells have 0 V, their respective alarm indicators have turned into red colour. We can conclude that this malfunction situation has been successfully detected and reported.

## 5. Conclusions

In this work, the design and development of a new, scalable and low cost multi-channel monitoring system for PEFCs has been reported. This system performs non-intrusive voltage measurements of each individual cell of a PEFC stack and it is scalable, in the sense that it is capable of carrying out measurements in stacks from 1 to 120 cells. Application to PEFC single cell voltage data gathering has allowed the experimental validation of the proposed system.

Some factors affect cell behaviour in the stack. Hence, cell voltage monitoring is a critical concern in the PEFC scope, so the designed system tries to contribute to such a challenging issue. An open-source Arduino MEGA2560 platform is used for data acquisition. A NI LabVIEW-based application performs data storage and representation in real-time. Such integration can be considered a novelty in the scientific literature for PEFC-based systems.

Additionally, an original amplifying and multiplexing board (AMB) has been designed and developed to act as interface between the PEFC individual cells and the Arduino input channel. It provides eight input channels, each one amplified by an instrumentation amplifier, and one output channel that is connected to an analog input of the Arduino. The digital outputs of the Arduino establish the address to select the input channel to be read. This setting allows one to multiply by eight the number of analog input channels of each Arduino analog input. For example, if a stack of 80 cells needs to be monitored, 10 AMB + 10 Arduino MEGA analog inputs will fulfill the requirement.

The monitoring platform is composed by a GUI where numerical and graphical data are displayed. User-configurable ability delivers some choices for data representation such as the number of samples for average value calculation, the sampling time and the cell voltage to be visualized. Such development has the advantages of low cost, easy to build and to maintain, scalability, versatility and flexible design.

Experimental results show reliable and accurate measurements in combination with proper data logging and visualization. In this sense, the presented prototype demonstrates the feasibility of the approach. Some future guidelines are now commented on. To begin with, the exposed procedure may serve as basis of a methodology to develop further monitoring systems including other critical parameters of PEFC operation. For instance, current, temperature, hydrogen flow, pressure and other magnitudes can be dealt.

Besides the continuous real-time monitoring of the PEFC voltage evolution, the registered data are intended to serve to deploy works related with elaboration of accurate models of PEFC, fault detection, state-of-health diagnostics and prognostics, low-level control, *etc*.

Furthermore, a master-slave structure to increase the sampling frequency for transient behaviour data registering is currently under development. Cloud storage of achieved data base will be an interesting issue to improve the system capabilities.

## Figures and Tables

**Table 1:** Main features of the PE stack.

Parameter	Value
Number of cells	10
Generated voltage	0.4–0.96 V per cell
Power	200 mW per cell
Electrode area	4 cm^2^ per cell
Weight	430 g
H × W × D	60 × 70 × 175 mm

## Abbreviations

The following abbreviations are used in this manuscript:

PE	Polymer Electrolyte
PEFC	Polymer Electrolyte Fuel Cell
DAQ	Data Acquisition
NI	National Instruments
BoP	Balance of Plant
RES	Renewable Energy Sources
SG	Smart Grid
PHM	Prognostics and Health Management
MTU	Master Terminal Unit
OPC	OLE for Process Control
SCADA	Supervisory Control And Data Acquisition
AMB	Amplifying and Multiplexing Board
IDE	Integrated Development Environment
VI	Virtual Instrument
GUI	Graphical User Interface
CSV	Comma Separated Values
